# Noninvasive and Individual‐Centered Monitoring of Uric Acid for Precaution of Hyperuricemia via Optical Supramolecular Sensing

**DOI:** 10.1002/advs.202104463

**Published:** 2022-04-28

**Authors:** Yaping Zhang, Huijuan Yu, Shiwei Chai, Xin Chai, Luyao Wang, Wen‐Chao Geng, Juan‐Juan Li, Yu‐Xin Yue, Dong‐Sheng Guo, Yuefei Wang

**Affiliations:** ^1^ State Key Laboratory of Component‐based Chinese Medicine Tianjin Key Laboratory of TCM Chemistry and Analysis Tianjin University of Traditional Chinese Medicine Tianjin 301617 China; ^2^ First Teaching Hospital of Tianjin University of Traditional Chinese Medicine National Clinical Research Center for Chinese Medicine Acupuncture and Moxibustion Tianjin 300193 China; ^3^ College of Chemistry Key Laboratory of Functional Polymer Materials (Ministry of Education) State Key Laboratory of Elemento‐Organic Chemistry Nankai University Tianjin 300071 China

**Keywords:** hyperuricemia, indicator displacement assay, noninvasive diagnosis, smartphone, uric acid

## Abstract

Characterized by an excessively increased uric acid (UA) level in serum, hyperuricemia induces gout and also poses a great threat to renal and cardiovascular systems. It is urgent and meaningful to perform early warning by noninvasive diagnosis, thus conducing to blockage of disease aggravation. Here, guanidinocalix[5]arene (GC5A) is successfully identified from the self‐built macrocyclic library to specifically monitor UA from urine by the indicator displacement assay. UA is strongly bound to GC5A at micromolar‐level, while simultaneously excluding fluorescein (Fl) from the GC5A·Fl complex in the “switch‐on” mode. This method successfully differentiates patients with hyperuricemia from volunteers except for those with kidney dysfunction and targets a volunteer at high risk of hyperuricemia. In order to meet the trend from hospital‐centered to individual‐centered testing, visual detection of UA is studied through a smartphone equipped with a color‐scanning feature, whose adaptability and feasibility are demonstrated in sensing UA from authentic urine, leading to a promising method in family‐centered healthcare style. A high‐throughput and visual detection method is provided here for alarming hyperuricemic by noninvasive diagnosis.

## Graphical abstract

A noninvasive and individual‐centered method is accomplished for monitoring uric acid (UA) in urine by executing indicator displacement assay, which successfully differentiates hyperuricemia patients from volunteers and targets a volunteer at high risk of hyperuricemia. To fit the family‐centered healthcare style, optical supramolecular sensing of UA is achieved by reading *G* value of images acquired with a portable smartphone.

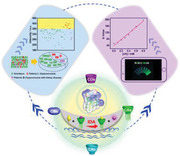

## 1 Introduction

Following hypertension, hyperlipidemia, and hyperglycemia, hyperuricemia has become one of the most common metabolic diseases,^[^ ^1^ ^]^ which is primarily caused by excessive elevation of serum uric acid (UA) owing to “underexcretion” or “renal overload” of the urate.^[^ ^2^, ^3^, ^4^ ^]^ Hyperuricemia is the most crucial risk factor for the occurrence of gout triggered by the deposition of monosodium urate crystals around the joints derived from the supersaturation of urate in the blood.^[^ ^5^, ^6^, ^7^ ^]^ Also, hyperuricemia greatly increases the patient's susceptibility to chronic renal insufficiency, cardiovascular diseases, and diabetes,^[^ ^8^, ^9^, ^10^ ^]^ resulting in reduced quality of life.^[^ ^11^, ^12^ ^]^ Therefore, it is of great significance to prevent and interrupt the pathophysiologic process of hyperuricemia by monitoring UA.

Generally, the clinical diagnosis of hyperuricemia is determined according to the blood UA level (>357.0 × 10^−6^ m for women and >416.5 × 10^−6^ m for men) by enzyme‐coupling method,^[^ ^13^, ^14^ ^]^ which requires invasive blood collection, specific instruments, and well‐trained hospital operators. Moreover, it can be a considerable challenge for the elderly and handicapped patients to accomplish the diagnosis. Nowadays, preventive medicine has entered the spotlight and attracted global attention, which will contribute to the economic utilization of medical resources and a great improvement in the quality of life for people. Accordingly, in order to realize the shift from hospital‐centered to family‐centered testing,^[^ ^15^ ^]^ there is an imperative need to develop a noninvasive, portable, accessible, and environment‐friendly method for daily monitoring based on the obvious fact that invasive blood collecting is not an ideal choice in family‐centered healthcare style.

As the most optimal research object, urine can be noninvasively collected and carries important physical and chemical information related to many metabolic diseases, such as diabetes, gouty arthritis, and renal disease diacrisis.^[^ ^16^, ^17^ ^]^ Many methods have been established to measure the UA level in urine, including absorption spectrophotometry,^[^ ^18^ ^]^ fluorescence biosensors,^[^ ^17^, ^19^ ^]^ electrochemical biosensors,^[^ ^20^, ^21^ ^]^ high‐performance liquid chromatography‐ultraviolet (HPLC‐UV),^[^ ^22^ ^]^ and HPLC‐mass spectrometry,^[^ ^23^ ^]^ all of which can be employed to monitor UA in urine with the merits of high sensitivity and excellent specificity to facilitate the diagnosis of hyperuricemia. Noticeably, the use of harmful reagents as mobile phase or solvent poses a threat to the environment and human health, which deviates from the concept of green development. What is important is that the reported methods exhibit obvious limitations which hindered the individual‐centered monitoring of UA, as evidenced by the high cost of employing such professional instruments together with the laborious and time‐consuming operation for sample pretreatment.

As a rapidly evolving frontier cross‐discipline, supramolecular chemistry sheds the light on an approach to monitor the analytes which surpasses the classic spectral and chromatographic methods. As a facile, environmentally friendly, and high‐throughput supramolecular strategy, indicator displacement assay (IDA) offers an alternative noncovalent approach for determination by molecular sensing in aqueous solution with advantages of simplicity, modularity, and tunability,^[^ ^24^ ^]^ enabling the translation of the microcosmic behavior of molecular recognition into the macroscopic signal of fluorescence. Recently, IDA has become prevalent, which utilizes specific cavity microenvironments to perform intermolecular interactions with analytes to facilitate the determination of diagnostic biomarkers.^[^ ^25^, ^26^ ^]^ In order to satisfy the diversified needs for monitoring analytes, supramolecular macrocycles, such as cyclodextrins (CDs),^[^ ^27^, ^28^ ^]^ cucurbit[n]urils (CBs),^[^ ^29^, ^30^, ^31^ ^]^ and calixarenes (CAs),^[^ ^32^, ^33^, ^34^ ^]^ were designed to produce different cavities, charged head bases, and hydrophobic alkyl chains. By executing IDA, the ideal biomarkers of thrombotic diseases (trimethylamine *N*‐oxide) and renal dysfunction (creatinine) were successfully detected in body fluids via supramolecular sensing with macrocycles.^[^ ^35^, ^36^ ^]^ Supramolecular sensing shows a promising prospect for application in family‐centered healthcare for disease diagnosis.^[^ ^37^, ^38^ ^]^

In our study, a noninvasive and individual‐centered method to monitor UA in urine was successfully performed by a supramolecular optical strategy. For accomplishing the detection of UA through implementing IDA, the key challenge is to design artificial receptors that are capable of strongly and selectively binding UA in the assay. From ourself‐built macrocyclic library, including CAs, CDs, and CBs, guanidinocalix[5]arene (GC5A), was screened based on the association ability between macrocycles and UA by employing fluorescein (Fl) as a fluorescent indicator. A “switch‐on” sensing of fluorescence was successfully undertaken for UA, from which a synchronous increase in fluorescence signal was witnessed along with increasing UA level. Excitingly, we successfully applied the established method to distinguish hyperuricemia patients from volunteers except for those with renal impairment. Meaningfully, one volunteer was identified as a high‐risk person. With accessible smartphones equipped with color‐scanning function, the improved method enabled visual sensing of UA in urine from volunteer and hyperuricemia patient, which offers a prospective access to an early warning of dangerous UA level for family‐centered healthcare.

## 2 Results and Discussion

### 2.1 Screening of UA Artificial Receptor from Macrocyclic Library

In the light of association constant (*K*_a_) of the indicator‐host and analyte‐host complexes, a competitive analyte can reversibly displace the indicator from the artificial receptor in an IDA, causing the “switch‐on” or “switch‐off” of the fluorescence signal.^[^ ^39^, ^40^ ^]^ Therefore, it is the prerequisite issue to screen UA artificial receptors from ourself‐established macrocyclic library for performing a successful assay of supramolecular sensing.

**Scheme 1:**
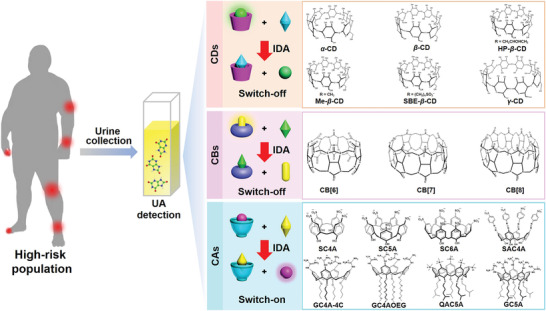
Schematic illustration for identifying UA artificial receptor from macrocyclic library by IDA.

In our macrocyclic library, seventeen macrocyclic receptors (**Scheme** **1**) were offered for the sensing of UA in 2‐[4‐(2‐hydroxyethyl)piperazin‐1‐yl]ethanesulfonic acid (HEPES) buffer solution (**Table** **1**), including CDs (*α*‐cyclodextrin, *α*‐CD; *β*‐cyclodextrin, *β*‐CD; (2‐hydroxypropyl)‐*β*‐cyclodextrin, HP‐*β*‐CD; sulfobutylether‐*β*‐cyclodextrin, SBE‐*β*‐CD; methyl‐*β*‐cyclodextrin, Me‐*β*‐CD; *γ*‐cyclodextrin, *γ*‐CD), CBs (cucurbit[6]uril, CB[6]; cucurbit[7]uril, CB[7]; cucurbit[8]uril, CB[8]), and CAs (*p*‐sulfonatocalix[4]arene, SC4A; *p*‐sulfonatocalix[5]arene, SC5A; *p*‐sulfonatocalix[6]arene, SC6A; sulfonated azocalix[4]arene, SAC4A; 5,11,17,23‐tetraguanidinium‐25,26,27,28‐tetrabutoxycalix[4]arene, GC4A‐4C; oligoethylene glycol functionalized guanidinocalixarene, GC4AOEG; 5,11,17,23,29‐penta(trimethylammonium)‐31,32,33,34,35‐penta (4‐methylpentloxy)calix[5]arene, QAC5A; GC5A).

**Table 1:** The association constants of reporter pairs between fluorescent dyes/UA and macrocycles

Reporter pair		*λ*_ex_ (*λ*_em_)^a)^ [nm]	*K*_a_ (host·dye) [M^–1^]	*K*_a_ (host·UA)^b)^ [M^–1^]
Macrocycle	Dye			
*β*‐CD	MB	664 (688)	(4.76 ± 0.21) × 10^2^	–
HP‐*β*‐CD	MB	664 (688)	(3.53 ± 0.06) × 10^2^	–
SBE‐*β‐*CD	NR	460 (573)	(1.59 ± 0.04) × 10^3^	–
Me‐*β*‐CD	NR	460 (573)	(6.87 ± 0.12) × 10^2^	–
*γ*‐CD	HPTS	405 (435)	(5.61 ± 0.28) × 10^[^ ^35^ ^]^	–
CB[6]	DSMI	450 (582)	(1.68 ± 0.26) × 10^5 [^ ^35^ ^]^	–
CB[7]	AO	450 (510)	(9.04 ± 0.54) × 10^4 [^ ^35^ ^]^	–
CB[8]	Me_2_DAP	335 (449)	(1.27 ± 0.42) × 10^6 [^ ^35^ ^]^	–
SC4A	LCG	368 (505)	(1.26 ± 0.13) × 10^7 [^ ^35^ ^]^	–
SC5A	LCG	368 (505)	(1.48 ± 0.21) × 10^6 [^ ^35^ ^]^	–
SC6A	LCG	368 (505)	(4.96 ± 0.62) × 10^7 [^ ^35^ ^]^	–
SAC4A	RhB	554 (576)	(9.26 ± 0.63) × 10^6^	–
GC4A‐4C	Fl	500 (513)	(1.31 ± 0.10) × 10^5 [^ ^33^ ^]^	–
GC4AOEG	EY	517 (537)	(2.37 ± 0.12) × 10^5 [^ ^55^ ^]^	–
QAC5A	EY	517 (537)	(2.00 ± 0.43) × 10^7^	–
GC5A	Fl	500 (513)	(5.0 ± 1.0) × 10^6 [^ ^33^ ^]^	(2.87 ± 0.23) × 10^5^

^a)^ *λ*_ex_ represents the wavelength of fluorescence excitation. *λ*_em_ represents the maximum wavelength of fluorescence emission;

^b)^ “−” represents no obvious binding detected.

As an eco‐friendly macrocycle, CDs are linked by *α*‐1,4‐glycosidic bonds with a hydrophilic external surface and a hydrophobic hollow cavity, conducing to the design of stimuli‐responsive supramolecular systems for the varied analytes.^[^ ^41^, ^42^ ^]^ As for the indicators always combined with CDs, six reporter pairs of CDs were usually employed, including *α*‐CD·*p*‐dimethylaminobenzonitrile (DMABN),^[^ ^43^ ^]^ *β*‐CD·methylene blue (MB),^[^ ^44^ ^]^ HP‐*β*‐CD·MB,^[^ ^44^ ^]^ SBE‐*β*‐CD·neutral red (NR),^[^ ^45^ ^]^ Me‐*β*‐CD·NR, and *γ*‐CD·8‐hydroxypyrene‐1,3,6‐trisulfonate (HPTS),^[^ ^46^ ^]^ each of which was tested in monitoring UA. Unfortunately, for the reporter pair of *α*‐CD·DMABN, it was proved to be infeasible because of the apparent interference with the fluorescence signal of DMABN by UA (Figure S1, Supporting Information). Meanwhile, no significant association was found between *α*‐CD and UA at 25 ℃ by microcalorimetric titration, which is displayed in Figure S2 in the Supporting Information. For other reporter pairs of CDs, fluorescence signals did not concomitantly decrease from the free indicators, implying that UA cannot expel the indicators from the cavities of CDs by competing against the cavities (Figures S3–S7, Supporting Information). Resembling the appearance of pumpkins, CBs feature two hydrophilic carbonylated rims and a hydrophobic cavity, which are endowed with the competence to encapsulate neutral and cationic guests such as metal ions, amines, and ammonium ions.^[^ ^30^, ^42^ ^]^ The classic reporter pairs were available in sensing UA, containing CB[6]·*trans*‐4‐[4‐(dimethylamino)styryl]‐1‐methylpyridinium iodide (DSMI),^[^ ^47^ ^]^ CB[7]·acridine orange (AO),^[^ ^48^ ^]^ and CB[8]·*N*,*N*’‐dimethyl‐2,7‐diazapyrenium (Me_2_DAP).^[^ ^49^ ^]^ By competitive fluorescence titrations, there was no binding toward UA obtained by the CBs hosts (Figures S8–S10, Supporting Information). Collectively, the incompatibility between the hydrophobic cavity microenvironment of hosts and hydrophilic UA (oil/water partition coefficients, −0.19) is responsible for occurrence of the insufficient inclusion complexation between UA and the hosts. Therefore, CDs and CBs failed to recognize UA.

From CDs and CBs, no macrocyclic host was perceived to effectively sense UA. Consequently, we turned our attention to the family of CAs described as “(almost) unlimited possibilities”.^[^ ^50^, ^51^ ^]^ CAs can be readily designed to obtain adjustable cavities and functionalized derivatives with various hydrophobic alkyl chains and charged head groups, which are endowed with the power to encapsulate substrates with different sizes, shapes, electric charges, and surface properties.^[^ ^33^, ^34^, ^35^, ^52^, ^53^ ^]^ *p*‐Sulfonatocalix[*n*]arenes (SC*n*As, *n* = 4, 5, 6) modified with multiple sulfonate head groups were employed to monitor UA with the lucigenin (LCG) as an indicator. In the competitive fluorescence titration, the fluorescence signals of the complexes did not exhibit remarkable changes as UA level increased, suggesting that SC*n*As are undesirable candidates for sensing UA (Figure S11, Supporting Information). SAC4A is a deep cavitand derivative of SC4A by the modification of azobenzene.^[^ ^54^ ^]^ We also failed to obtain the binding affinity of SAC4A to UA by employing SAC4A·rhodamine B (RhB) as the reporter pair (Figure S12, Supporting Information). Apart from different hydrophobic alkyl chains at the lower rim, C4A and C5A were decorated with quaternary ammonium or guanidine at the upper rim to yield GC4A‐4C,^[^ ^33^ ^]^ GC4AOEG,^[^ ^55^ ^]^ QAC5A, and GC5A, allowing for the diversified dimensions of cavities and the ability for the formation of charge‐assisted hydrogen bonds (salt bridge). However, GC4A‐4C, GC4AOEG, and QAC5A were incapable of affording UA, leading to the unsatisfactory association constant detected by executing IDA with Fl, eosin Y (EY), and EY as the indicators, respectively (Figures S13–S15, Supporting Information). It is reassuring to note that the dimension of the GC5A cavity was tolerable for UA and the guanidine group was empowered to potentially interact with UA via electrostatic and hydrogen bonds. Hence, the fluorescence of Fl was excited to show a concentration‐dependent increase with elevating UA level, which would qualify to sense UA.

### 2.2 Investigation of the GC5A·UA Complex by Theoretical Calculation

**Figure 1:**
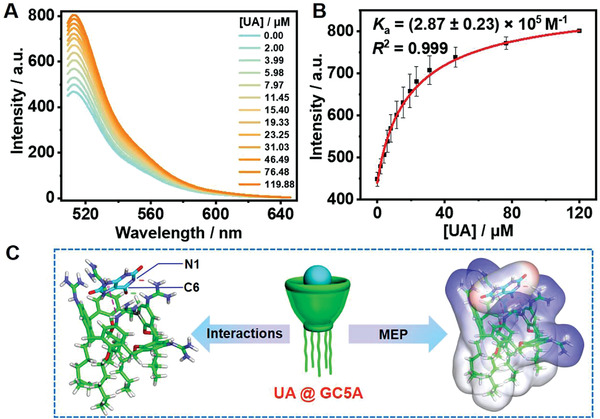
Competitive titration in the GC5A·Fl complex by UA and MD simulation of GC5A·UA. A) Competitive titration in the GC5A·Fl (0.80 × 10^−6^/1.00 × 10^−6^ m) reporter pair performed with UA up to 119.88 × 10^−6^ m at *λ*_ex_ = 500 nm and *λ*_em_ = 513 nm (*n *= 3). B) Competitive titration curve acquired according to a 1:1 competitive binding model in HEPES buffer solution (10 × 10^−3^ m, pH = 7.4) at 25 °C (*n *= 3). C) Interactions and corresponding MEP in the GC5A·UA complex by a 100 ns MD simulation. Electrostatic, hydrogen bonding, and *π*−cation interactions were respectively colored by red, green, and pink dotted lines, in which C atoms of host and guest were respectively shown in green and cyan.

On account of the screening results for UA artificial receptor from macrocyclic library, the GC5A·Fl reporter pair was selected in fluorescence “switch‐on” mode, which was validated to possess a desirable association constant (*K*_a_ = (5.0 ± 1.0) × 10^6^ m ^−1^) by the fluorescence titration and UV–vis titration.^[^ ^33^, ^35^ ^]^ Moreover, UA did not interfere with the fluorescence intensity of Fl in the range of 0–447.08 × 10^−6^ m (Figure S13A, Supporting Information). As shown in **Figure** **1**A, UA can competitively associate with GC5A against Fl in a concentration‐dependent manner, indicating the observation of a relatively strong binding affinity ((2.87 ± 0.23) × 10^5^ m ^−1^) between UA and GC5A (Figure 1B).

The complexation of UA with GC5A was further confirmed by a 100 ns molecular dynamics (MD) simulation (Figure 1C). From the 100 ns MD simulation, it is evident that UA was embedded into the cavity of GC5A formed by the upper rim. Electrostatic interaction was found between N1 of UA and hydrogen atom from the guanidine group of GC5A. Furthermore, as a common noncovalent interaction, hydrogen bonding is considered to take place based on the angle C−H···O > 135° and the distance C···O < 3.5 Å. From GC5A, the hydrogen atom of the guanidinium group was attached to oxygen atom at C6 of UA, resulting in the formation of N—H···O hydrogen bond. In addition, *π*−cation interaction was also found. What is more, the molecular electrostatic potential (MEP) was mapped on the molecular van der Waals surfaces of GC5A and UA. A complementary manner of the MEP was witnessed between the positively charged guanidine group (blue areas) of GC5A and the negatively charged oxygen atoms (red areas) of UA in the GC5A·UA complex. Collectively, there are electrostatic, hydrogen bonding, *π*−cation interactions, and MEP that promoted the stable binding between GC5A and UA, contributing to the satisfactory performance for UA sensing by the GC5A·Fl reporter pair.

### 2.3 Study on the Specific Detection of UA from Urine by the GC5A·Fl Complex

**Figure 2:**
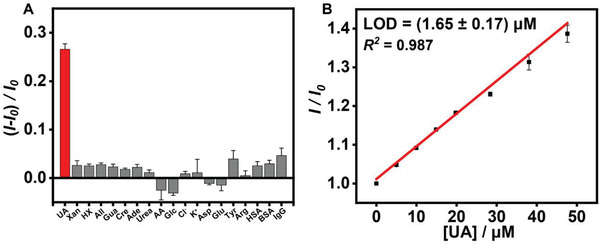
Specificity and sensitivity for UA sensing. A) Fluorescence responses of the GC5A·Fl (0.80 × 10^−6^/1.00 × 10^−6^ m) reporter pair at *λ*_ex_ = 500 nm and *λ*_em_ = 513 nm upon addition of UA or endogenous substances in HEPES buffer solution (10 × 10^−3^ m, pH = 7.4) at 25 °C, whose concentration was 0.40 mg L^−1^ for BSA and HSA, 5 mg L^−1^ for IgG, 3 × 10^−3^ m for Glc, 0.30 × 10^−3^ m for urea, Cl^–^, K^+^, Tyr, Arg, Asp, Glu, and AA, and 10 × 10^−6^ m for UA, Xan, HX, All, Ade, Gua, and Cre, respectively (*n* = 3). *I*_0_ and *I* are the fluorescence intensities of the GC5A·Fl complex (0.80 × 10^−6^/1.00 × 10^−6^ m) in the absence and presence of the tested analytes, respectively. B) The correlation between fluorescence response and the increased level of UA in the tenfold diluted artificial urine at 25 °C (*n* = 3). *I*_0_ and *I* are the fluorescence intensities of the GC5A·Fl complex (8.00 × 10^−6^/4.00 × 10^−6^ m) in the absence and presence of the tested analytes, respectively.

**Figure 3:**
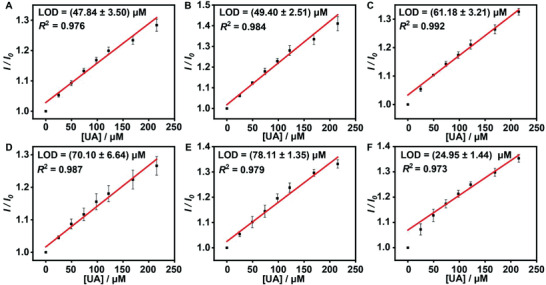
The calibration curves and LOD of UA acquired in the 50‐fold diluted urine from different volunteers (A–F) at 25 °C (*n* = 3). *I*_0_ and *I* are the fluorescence intensities of the GC5A·Fl complex (10.00 × 10^−6^/5.00 × 10^−6^ m) before and after addition of UA.

As shown in Figure S16 in the Supporting Information, there was a linear increase of fluorescence (*R* ^2^ = 0.988) excited by the extruded Fl from the GC5A·Fl complex (0.80 × 10^−6^/1.00 × 10^−6^ m) accompanied by the increasing UA level in HEPES buffer solution. The low detection limit (LOD) of UA was calculated to be 1.53 × 10^−6^ m using the 3*σ*/slope method.^[^ ^56^, ^57^ ^]^ In order to investigate the sensing specificity for UA by GC5A, typical endogenous substances in urine were added into the GC5A·Fl complex (0.80 × 10^−6^/1.00 × 10^−6^ m), including xanthine (Xan), hypoxanthine (HX), allopurinol (All), guanine (Gua), creatinine (Cre), adenine (Ade), urea, ascorbic acid (AA), glucose (Glc), Cl^–^, K^+^, aspartic acid (Asp), glutamic acid (Glu), tyrosine (Tyr), arginine (Arg), human serum albumin (HSA), bovine serum albumin (BSA), and immunoglobulin G (IgG), respectively. The fluorescent interference caused by endogenous substances was negligible compared to the fluorescence response triggered by UA (**Figure** **2**A). Given that these endogenous substances may bind to UA and further affect the UA sensing, UA and endogenous substances were concurrently titrated into the GC5A·Fl complex (0.80 × 10^−6^/1.00 × 10^−6^ m), including UA+HSA, UA+IgG, UA+Xan, UA+HX, UA+Arg, UA+K^+^, and UA+Glc, respectively. The addition of endogenous substances did not lead to a significant variation on the fluorescence response caused by UA (Figure S17, Supporting Information). Furthermore, no appreciable influence was observed on the fluorescence intensity of Fl by the cumulative addition of the endogenous substances in the measured range (Figure S18, Supporting Information). Also, the sensitive detection of UA by the GC5A·Fl reporter pair was demonstrated by gradually titrating UA into a tenfold diluted artificial urine, showing an acceptable correlation (*R* ^2^ = 0.987) between fluorescence response and UA level (0–47.64 × 10^−6^ m), as displayed in Figure 2B. The linearity was successfully validated by the same procedure in 50‐fold diluted urine from different volunteers over the tested range of UA (0–215.31 × 10^−6^ m) (**Figure** **3**). From Figures 2B and 3, as is evident, the LOD was recorded down to 1.65 × 10^−6^ ± 0.17 × 10^−6^ m in the artificial urine and 24.95 × 10^−6^ ± 1.44 × 10^−6^ m in the urine from volunteers, respectively.

### 2.4 Differentiation of UA Level in Urine between Volunteers and Hyperuricemia Patients

**Figure 4:**
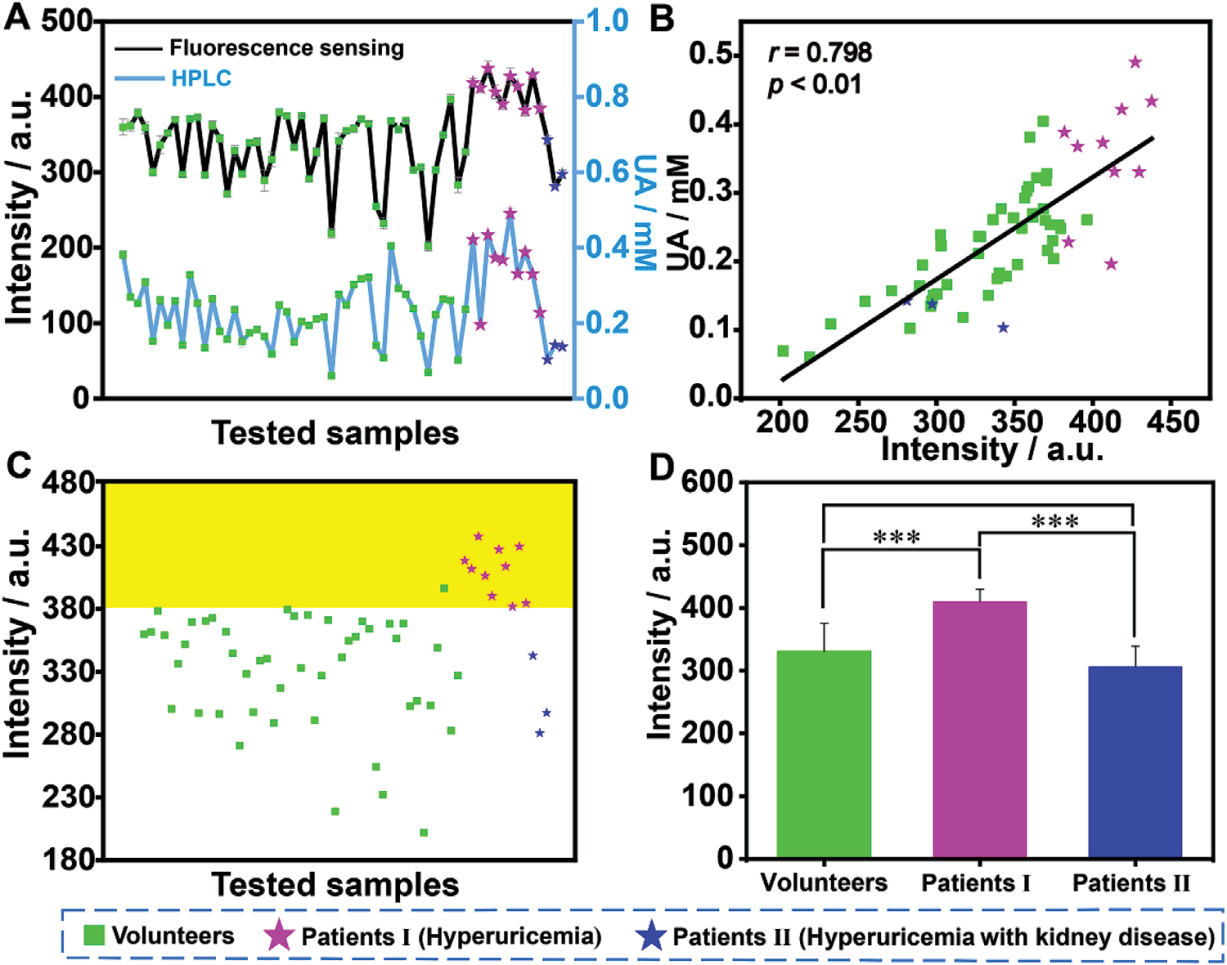
Applicability of fluorescence sensing for UA monitoring and UA level in urine from volunteers and patients. A) The determined results of UA in urine from patients and volunteers by fluorescence sensing and HPLC (*n* = 3). B) A moderately positive correlation (*r* = 0.798, *p* < 0.01) of UA level determined by HPLC and fluorescence assay in urine. C) Fluorescence intensity of the GC5A·Fl complex (10.00 × 10^−6^/5.00 × 10^−6^ m) at *λ*_ex_ = 500 nm and *λ*_em_ = 513 nm exposed to UA in the 50‐fold diluted urine from patients and volunteers (*n* = 3). D) Statistical analysis by Student's *t*‐test among the groups of volunteers, hyperuricemia patients, and hyperuricemia patients with kidney disease (***, *p* < 0.001).

To evaluate practicability and applicability for sensing UA, the established method was tested by the GC5A·Fl reporter pair to monitor UA in the urine from volunteers, hyperuricemia patients, and hyperuricemia patients with kidney dysfunction, whose fluorescence intensities ranging from 201.99 to 437.65 (**Figure** **4**A). Furthermore, the classic HPLC method (Figure S19, Supporting Information) was adopted to demonstrate the reliability of the fluorescence method, by which UA was detected in the tested urine within the range of 0.78–200.00 mg L^–1^. As a result, as shown in Figure 4B, a positive correlation (*Pearson correlation coefficient*, *r* = 0.798, *p* < 0.01) was revealed between fluorescence and the HPLC method. Evidently, according to the fluorescence response (Figure 4C), it can be finely differentiated among volunteers (25 ± 2 years old) except for a man (25 years old) with abnormal UA level, hyperuricemia patients (49 ± 15 years old), and hyperuricemia patients with kidney insufficiency (73 ± 10 years old). The volunteer with abnormal UA level was further diagnosed as hyperuricemia clinically through classic enzyme linked assay, depending on the serum UA concentrations at 484.76× 10^−6^ and 510.50 × 10^−6^ m on different days. In the meantime, five volunteers with normal fluorescence intensities were randomly selected to detect UA level in clinics, where their serum UA level was within the normal range. Therefore, it suggests that the fluorescence method is simple, rapid, and predictable for the early diagnosis of hyperuricemia/gout by noninvasive monitoring of UA in urine.

As shown in Figure 4D, fluorescence intensities show a significant difference (*p* < 0.001) between the volunteers (330.89 ± 44.71) and hyperuricemia patients. Also, it is a substantial difference (*p* < 0.001) in fluorescence signals from hyperuricemia patients (410.20 ± 19.37) and hyperuricemia patients with kidney dysfunction (307.11 ± 31.96). However, no discrepancy in fluorescence signals (*p* > 0.05) was witnessed between hyperuricemia patients with kidney dysfunction and volunteers, revealing that the kidney, an important excretion organ, would suffer from incapacitation to remove harmful metabolites from the body through urine with dysfunction. Obviously, UA level in the blood mostly depends on the balance between its production and excretion. Overproduction and/or underexcretion will lead to hyperuricemia.^[^ ^58^ ^]^ Prolonged hyperuricemia induces progressive gout with joint damage and chronic synovitis,^[^ ^59^ ^]^ which also threatens kidney function severely.^[^ ^60^, ^61^ ^]^ This is how an unfortunate vicious circle starts. Therefore, as an early‐warning method, there exists an urgent demand for UA family‐centered and daily detection, which will facilitate the precaution of hyperuricemia.

### 2.5 Optical Supramolecular Sensing of UA with Smartphone

Recently, a widespread outbreak of chronic diseases has further constrained limited medical resources, triggering increased overcrowding in the hospital and uncomfortable experiences of patients. Fortunately, with the popularization of smartphones and various applications, individual‐centered healthcare has come true. Inspired by the successful application of real‐time/on‐site monitoring of environmental pollutants and biological molecular,^[^ ^32^, ^62^ ^]^ we succeeded in performing the optical supramolecular sensing of UA with a smartphone equipped with a color‐scanning feature.

**Figure 5:**
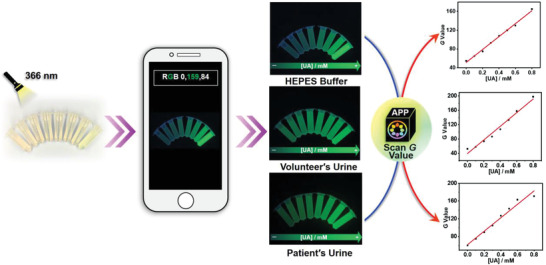
Visual monitoring of UA in HEPES buffer solution and urine from volunteer and hyperuricemia patient by smartphone with the GC5A·Fl reporter pair.

As shown in **Figure** **5**, by exposing UA to the GC5A·Fl complex (10.00 × 10^−6^/5.00 × 10^−6^ m), no marked difference is visually observed under daylight in a series of HEPES buffer solution with varying concentrations of UA (0–0.8 × 10^−3^ m) added. The phenomenon that intrigued us was that as soon as the samples were excited by ultraviolet (366 nm), the response of green fluorescence (*G* values) increased linearly in a UA concentration‐dependent pattern. Alternatively, same phenomena were witnessed in 200‐fold diluted urine from volunteer and hyperuricemia patient, which was further titrated using different concentrations of UA (0–0.8 × 10^−3^ m). The additional UA was added into urine samples from volunteer and hyperuricemia patient to demonstrate the linear relationship between the UA level and the *G* value even in practical urine samples, which is informative for quantitative detection. This portable, noninvasive strategy enables the implementation of home‐based UA optical supramolecular sensing.

## 3 Conclusion

In conclusion, from our self‐built macrocyclic library, the artificial receptor, GC5A, was identified to selectively and sensitively sense UA by employing Fl as an indicator in fluorescence “switch‐on” mode. Successful UA sensing was accomplished in artificial urine and authentic urine from human. Taking advantage of the specificity and reliability, we applied the established IDA method to distinguish volunteers from hyperuricemia patients with the exception of those suffering from hyperuricemia with kidney dysfunction, implicating that the excretory function of kidney plays an indispensable role in balancing the UA level in the blood. To accommodate the family‐centered healthcare style, optical supramolecular sensing of UA was exploratively carried out by reading the *G* value of images with a portable smartphone. Collectively, this work offers a rapid and sensitive strategy for sensing UA in urine through noninvasive diagnosis, providing a promising access to home‐based UA optical supramolecular sensing in our daily life.

## 4 Experimental Section

### Materials

UA, Fl, EY, DSMI, HPTS, LCG, AO, RhB, *α*‐CD, *β*‐CD, Me‐*β*‐CD, HP‐*β*‐CD, SBE‐*β*‐CD, *γ*‐CD, CB[6], CB[7], CB[8], Xan, HX, Cre, HSA, IgG, and methanol were purchased from Sigma‐Aldrich Co., Ltd. (St. Louis, Missouri, USA). HEPES and NR were provided by Tianjin Heowns Biochemical Technology Co., Ltd. (Tianjin, China). Glu, Asp, Tyr, Arg, Glc, and AA were manufactured by Yuanye Bio‐Technology Co., Ltd. (Shanghai, China). BSA was obtained from Beijing solarbio science and technology Co., Ltd. (Beijing, China). MB was offered by Merck KGaA Co., Ltd. (Darmstadt, Germany). Urea, potassium chloride, sodium chloride, potassium chloride, and sodium phosphate (monobasic) were bought from Tianjin Fengchuan Chemical Reagent Technology Co., Ltd. (Tianjin, China). Triethylamine was procured from Aladdin Biochemical Technology Co., Ltd. (Shanghai, China). Phosphoric acid was acquired from Tianjin Jinke Fine Chemical Industry Research Institute (Tianjin, China). Me_2_DAP was given as a gift from Prof. Frank Biedermann from Institute of Nanotechnology, Karlsruhe Institute of Technology (Eggenstein‐Leopoldshafen, Germany). GC5A, QAC5A, SAC4A, GC4A‐4C, GC4AOEG, SC4A, SC5A, and SC6A were synthesized as previously reported.^[^ ^33^, ^54^, ^55^, ^63^, ^64^, ^65^ ^]^

### Samples

HEPES buffer solution (10 × 10^−3^ m, pH = 7.4) was obtained by dissolving HEPES (2.38 g) in ultrapure water (1 L) at 25 °C. The HEPES buffer solution was further verified to pH 7.4 with sodium hydroxide (0.1 m). Artificial urine solution was prepared as previously reported.^[^ ^35^, ^66^ ^]^

Written informed consent was signed by 47 volunteers (25 ± 1 years old) and 13 patients (55 ± 18 years old). The acquired urine samples were stored at −80 °C before analysis. All urine specimens were collected from the midstream of morning urine with the corresponding supernatant collected after 15 min centrifugation (12 700 rpm) prior to analysis.

### Isothermal Titration Calorimetry (ITC)

The ITC measurements were undertaken by a fully computer‐operated isothermal calorimetry instrument (PEAQ‐ITC, Malvern). All titrations were performed in HEPES buffer solution (10 × 10^−3^ m, pH = 7.4) at 25 °C under atmospheric pressure. From a 40.0 µL syringe, a constant volume (2.0 µL per injection) of the UA solution (500 × 10^−6^ m) was injected into the reaction cell (280 µL) filled with *α*‐CD solution (50 × 10^−6^ m) within 4 s under stirring (750 rpm). Eighteen successive injections were conducted to record the microcalorimetric titration curve of the complexation of host·guest.

### Fluorescence Spectroscopy

The steady‐state fluorescence spectra were recorded in a conventional 10 × 10 × 45 mm^3^ quartz cell (light path = 10 mm) by Varian Cary Eclipse spectrometer (Agilent Technologies Inc., USA) equipped with a single‐cell peltier accessory. In order to obtain the association constant (*K*_a_), data were fitted by a host–guest binding stoichiometry (1:1) in the direct host–guest titrations.^[^ ^67^, ^68^ ^]^ From the competitive titrations, the data fitting was nonlinearly conducted by a 1:1 competitive binding model. All experiments were carried out in HEPES buffer solution (10 × 10^−3^ m, pH = 7.4) at 25 °C. The calibration lines were established at 25 °C in the light of the fluorescence responses of GC5A·Fl reporter pair upon gradual titration of UA in HEPES buffer solution, artificial urine solution, and urine from volunteers, respectively.

The experiments were repeated three times in this study with the results expressed as$\bar{x}\pm \mathrm{SD}$ (standard deviation, *n* = 3). Data were fitted in a nonlinear manner for direct host·guest titrations and competitive titrations,^[^ ^67^ ^]^ by using the fitting modules downloaded from the website of Prof. Nau's group (http://www.jacobs‐university.de/ses/wnau) under the column of “Fitting Functions”.

### Statistical Analysis

Data obtained from this study were used without preprocessing unless otherwise stated. Data were expressed as the mean ± SD for three independent experiments. The difference between two groups was analyzed using Student's *t*‐test. Significance was indicated by asterisks: ***p < 0.001. Pearson correlation analysis was conducted to test the correlation.

The Originpro 2016 software (Originlab Corp. Northampton, MA, USA) and SPSS 21.0 software (International Business Machines, Armonk, NY, USA) were used for the statistical analysis. All figures were drawn by Originpro 2016 software (Originlab Corp. Northampton, MA, USA) and Adobe Illustrator CC2020 (Adobe Systems Incorporated, San Jose, CA, USA).

### Theoretical Calculations for GC5A·UA Complex

The scalable program nanoscale molecular dynamics (NAMD) 2.14^[^ ^69^ ^]^ with the CHARMM36 force field^[^ ^70^, ^71^, ^72^ ^]^ and the TIP3P water model^[^ ^73^ ^]^ were employed to perform MD simulation. With the visual molecular dynamics (VMD) program (VMD 1.9.3, University of Illinois at Urbana‐Champaign), visualization and analysis of all the MD trajectories were carried out.^[^ ^74^ ^]^ By adding four Cl^–^ ions, the GC5A·UA complex was overall charge neutrality. Before simulation, the system (cubic box ≈ 46 × 49 × 45 Å^3^, total 9447 atoms) underwent 5000 steps minimization and 100 ps MD simulation with host·guest restrained, and 2 ns water equilibration without restrained. Then, the interaction between UA and GC5A was systematically investigated by performing a 100 ns MD simulation.

### HPLC Method

Quantification of UA in urine from volunteers and hyperuricemia patients was executed on an LC‐20AT HPLC system (Shimadzu, Japan). After deproteinization with methanol (1:1), all urine samples were diluted fivefold by 50% methanol aqueous solution (v/v), which were centrifuged for 15 min at 12 700 rpm. The assay was performed in triplicate for all samples. A series of UA standard solutions with different concentrations were employed for the construction of the calibration curve.

0.1% Phosphoric acid aqueous solution with 0.05% triethylamine (v/v) (A) and methanol (B) were applied for excellent separation of UA on a Diamonsil C18 column (4.6 mm × 250 mm, 5 µm) at 30 °C. The gradient program was undertaken as follows: 5% B in 0–4 min and 5–30% B in 4–13 min. The flow rate and injection volume were 1 mL min^–1^ and 10 µL, respectively.

### Optical Supramolecular Sensing UA with Smartphone

The GC5A·Fl (10.0 × 10^−6^/5.0 × 10^−6^ m) reporter pair was employed to sense UA (0–0.8 × 10^−3^ m) in HEPES buffer solution (10 × 10^−3^ m, pH = 7.4), and the 200‐fold diluted urine from volunteer and hyperuricemia patient, respectively. Sample solutions in 2.0 mL centrifuge tubes (Axygen Scientific, 2.0 mL) were excited with ultraviolet (366 nm) in thin layer chromatography scanner (DKSH Business Co., Ltd., China). Then, the excited fluorescence from samples was scanned by an iPhone XR (Apple, Inc., USA), which was processed by a color scanning application from Apple Store (Color Name, Vlad Polyanskiy).

## Conflict of Interest

The authors declare no conflict of interest.

## Author Contributions

Y.Z., H.Y., and S.C. contributed equally to this work. Y.Z., H.Y., D.‐S.G., and Y.W. conceived the experiment and designed the study. S.C. collected urine samples from patients in the hospital. W.‐C.G. and J.‐J.L synthesized host macrocycles. W.‐C.G. performed the theoretical calculation. Y.Z., H.Y., S.C., and Y.‐X.Y. performed all the other experiments. S.C., X.C., and L.W. investigated the project and analyzed data. Y.Z., H.Y., W.‐C.G., D.‐S.G., and Y.W. contributed to writing of this paper and all authors commented on it.

## Supporting information

Supporting Information

## Data Availability

The data that support the findings of this study are available from the corresponding author upon reasonable request.
