# Supplementary material for: Noninvasive and Individual‐Centered Monitoring of Uric Acid for Precaution of Hyperuricemia via Optical Supramolecular Sensing
Source: Adv Sci (Weinh). 2022 Apr 28;9(18):2104463. doi: 10.1002/advs.202104463 (PMC9218761; doi:10.1002/advs.202104463)
Supplement: Supplementary file 1 — Supporting Information [file ADVS-9-2104463-s001.pdf]

ADVANCED SCIENCE
Open Access

Supporting Information

for *Adv. Sci.*, DOI 10.1002/advs.202104463

Noninvasive and Individual-Centered Monitoring of Uric Acid for Precaution of Hyperuricemia via Optical Supramolecular Sensing

Yaping Zhang, Huijuan Yu, Shiwei Chai, Xin Chai, Luyao Wang, Wen-Chao Geng, Juan-Juan Li, Yu-Xin Yue, Dong-Sheng Guo and Yuefei Wang**

Supporting Information

Non-invasive and individual-centered monitoring of uric acid for precaution of hyperuricemia via optical supramolecular sensing

Yaping Zhang, Huijuan Yu, Shiwei Chai, Xin Chai, Luyao Wang, Wen-Chao Geng, Juan-Juan Li, Yu-Xin Yue, Dong-Sheng Guo and Yuefei Wang**

Y. Zhang, H. Yu, X. Chai, L. Wang, Prof. Y. Wang
State Key Laboratory of Component-based Chinese Medicine
Tianjin Key Laboratory of TCM Chemistry and Analysis
Tianjin University of Traditional Chinese Medicine
Tianjin 301617, China
E-mail: wangyf0622@tjutcm.edu.cn

W.-C. Geng, J.-J. Li, Y.-X. Yue, Prof. D.-S. Guo
College of Chemistry
Key Laboratory of Functional Polymer Materials (Ministry of Education)
State Key Laboratory of Elemento-Organic Chemistry
Nankai University
Tianjin 300071, China
E-mail: dshguo@nankai.edu.cn

S. Chai
First Teaching Hospital of Tianjin University of Traditional Chinese Medicine, National Clinical Research Center for Chinese Medicine Acupuncture and Moxibustion
Tianjin 300193, China

Y.Z., H.Y., and S.C. contributed equally to this work.

Supplementary Figures

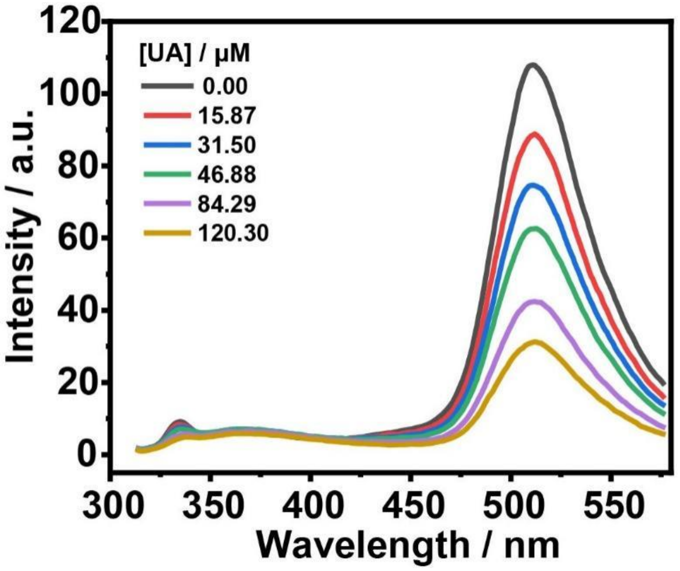

Figure S1. Fluorescence spectra of DMABN with UA in HEPES buffer solution (10 mM, pH = 7.4). Direct fluorescence titration of DMABN (10.00 μM) with UA up to 120.30 μM at λ_{ex} = 300 nm and λ_{em} = 525 nm in HEPES buffer solution at 25 °C.

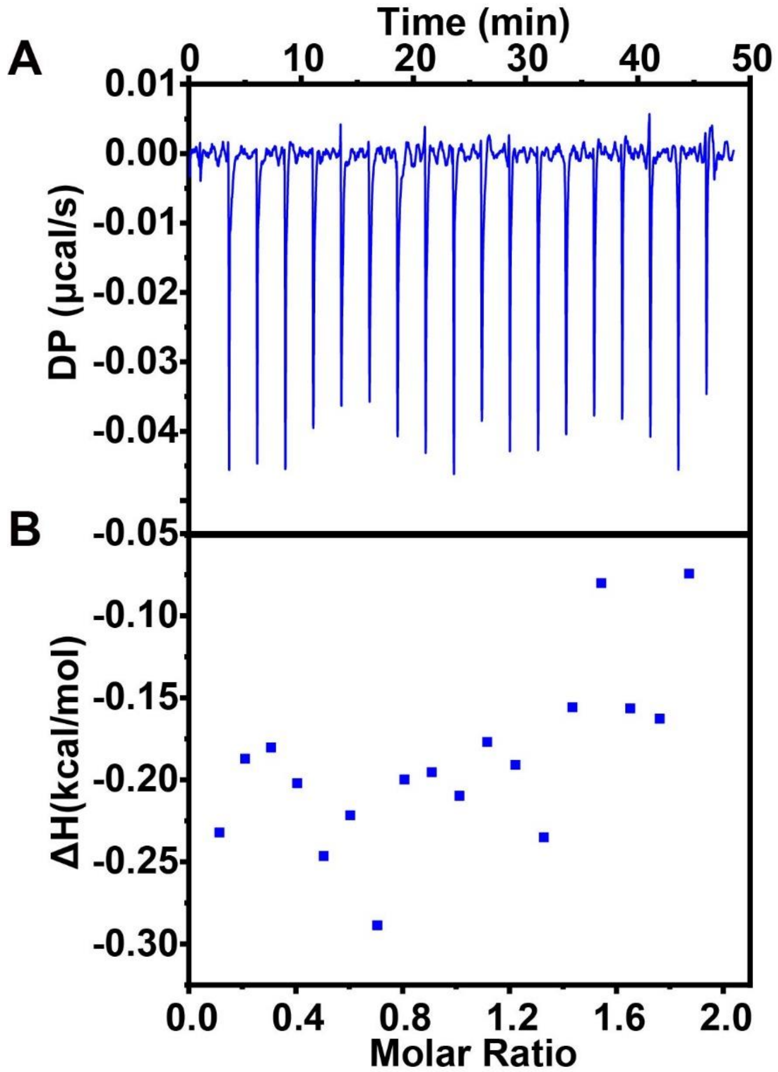

Figure S2. Microcalorimetric titration of α-CD (50 μM) with UA (500 μM) in HEPES buffer solution (10 mM, pH = 7.4) at 25 °C. (A) Raw ITC data from eighteen sequential injections (2 μL UA solution per injection) into α-CD. (B) The reaction heat obtained from integration of calorimetric trace.

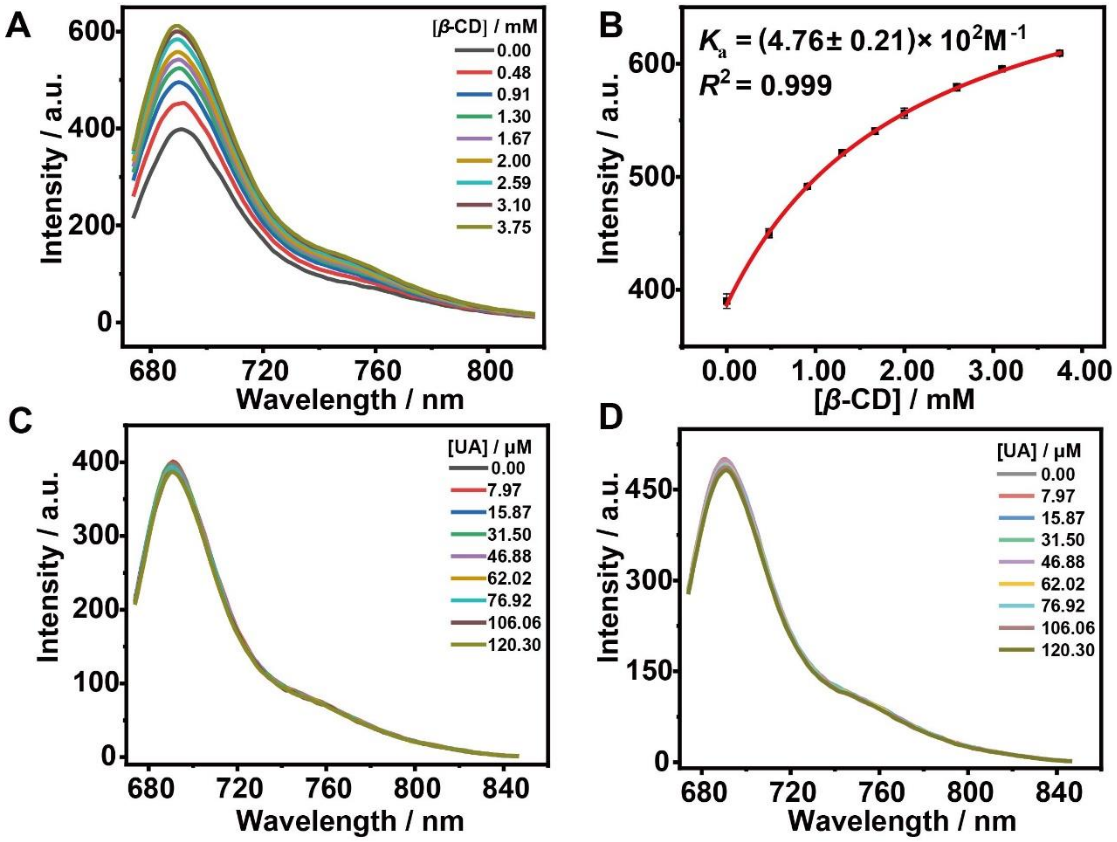

Figure S3. Direct fluorescence titration of MB with *β*-CD, fluorescence spectra of MB with UA, and competitive titration of the *β*-CD•MB reporter pair with UA in 10 mM HEPES buffer solution (pH = 7.4, 25 °C) at λ_{ex} = 664 nm and λ_{em} = 688 nm. (A) Direct fluorescence titration of MB (10.00 μM) with *β*-CD up to 3.75 mM. (B) The titration curve at λ_{em} = 688 nm acquired by a 1:1 binding model. (C) Fluorescence spectra of MB (10.00 μM) by adding UA up to 120.30 μM. (D) Competitive titration of the *β*-CD•MB (1.00 mM/10.00 μM) reporter pair with UA up to 120.30 μM.

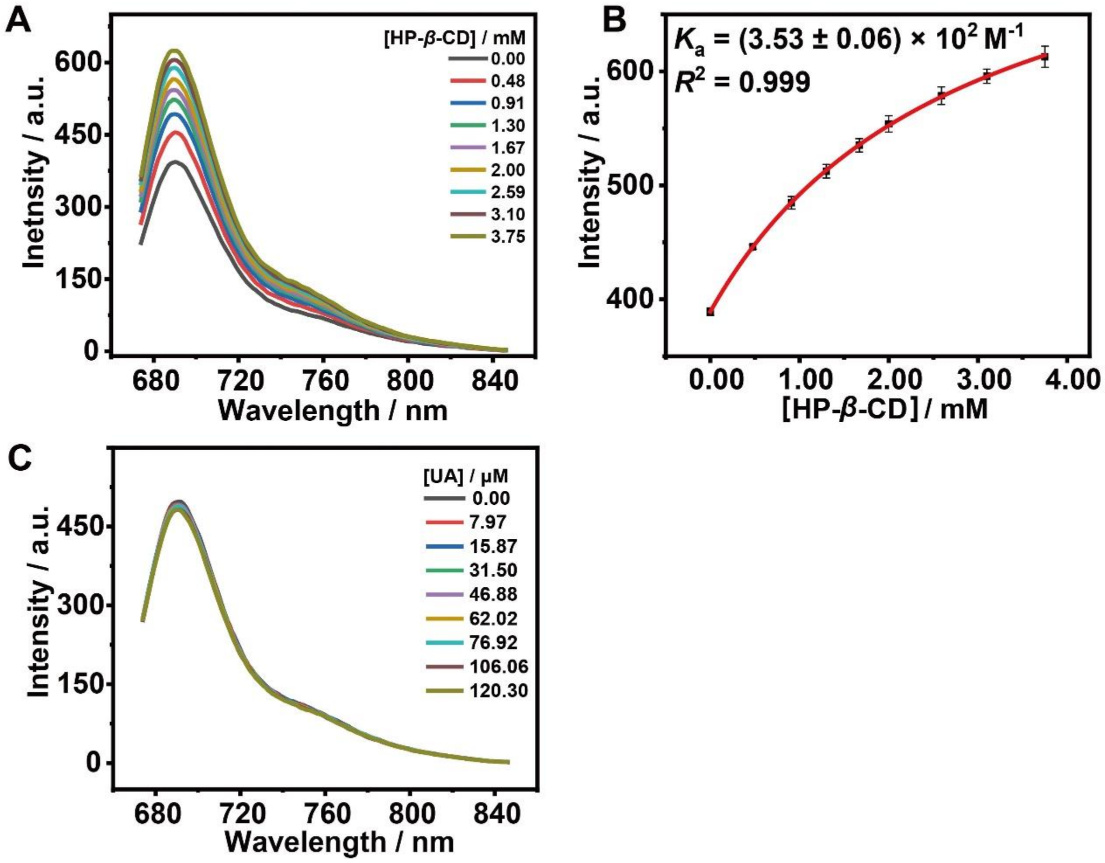

Figure S4. Direct fluorescence titration of MB with HP-*β*-CD and competitive titration of the HP-*β*-CD•MB reporter pair with UA in 10 mM HEPES buffer solution (pH = 7.4, 25 °C) at λ_{ex} = 664 nm and λ_{em} = 688 nm. (A) Direct fluorescence titration of MB (10.00 μM) with HP-*β*-CD up to 3.75 mM. (B) The titration curve at λ_{em} = 688 nm derived from a 1:1 binding model. (C) Competitive titration of the HP-*β*-CD•MB (1.00 mM/10.00 μM) reporter pair with UA up to 120.30 μM.

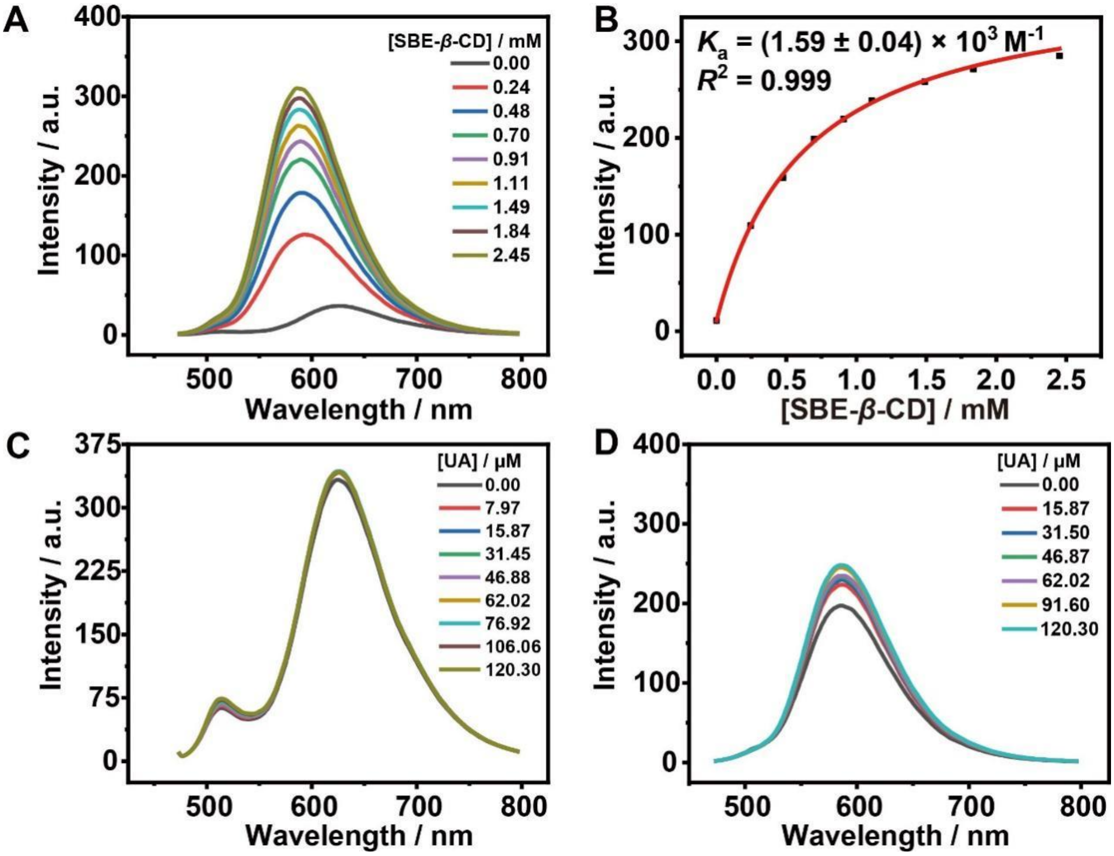

Figure S5. Direct fluorescence titration of NR with SBE-β-CD, fluorescence spectra of NR with UA, and competitive titration of the SBE-β-CD•NR reporter pair with UA in 10 mM HEPES buffer solution (pH = 7.4, 25 °C) at λ_{ex} = 460 nm and λ_{em} = 573 nm. (A) Direct fluorescence titration of NR (10 μM) with SBE-β-CD up to 2.45 mM. (B) The titration curve at λ_{em} = 573 nm gained from a 1:1 binding model. (C) Fluorescence spectra of NR (10 μM) by adding UA up to 120.30 μM. (D) Competitive titration of the SBE-β-CD•NR (1 mM /10 μM) reporter pair with UA up to 120.30 μM.

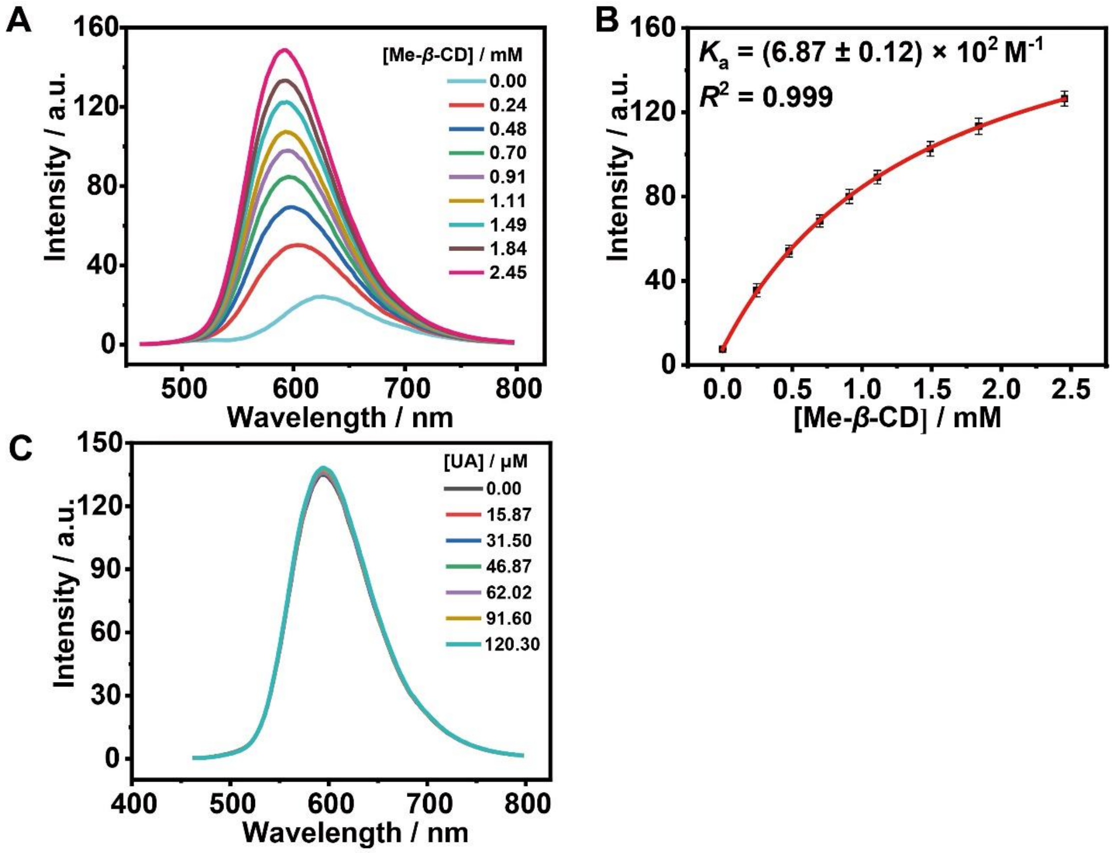

Figure S6. Direct fluorescence titration of NR with Me-β-CD and competitive titration of the Me-β-CD•NR reporter pair with UA in 10 mM HEPES buffer solution (pH = 7.4, 25 °C) at λ_{ex} = 460 nm and λ_{em} = 573 nm. (A) Direct fluorescence titration of NR (10 μM) with Me-β-CD up to 2.45 mM. (B) The titration curve at λ_{em} = 573 nm achieved by a 1:1 binding model. (C) Competitive titration of the Me-β-CD•NR (1 mM /10 μM) reporter pair with UA up to 120.30 μM.

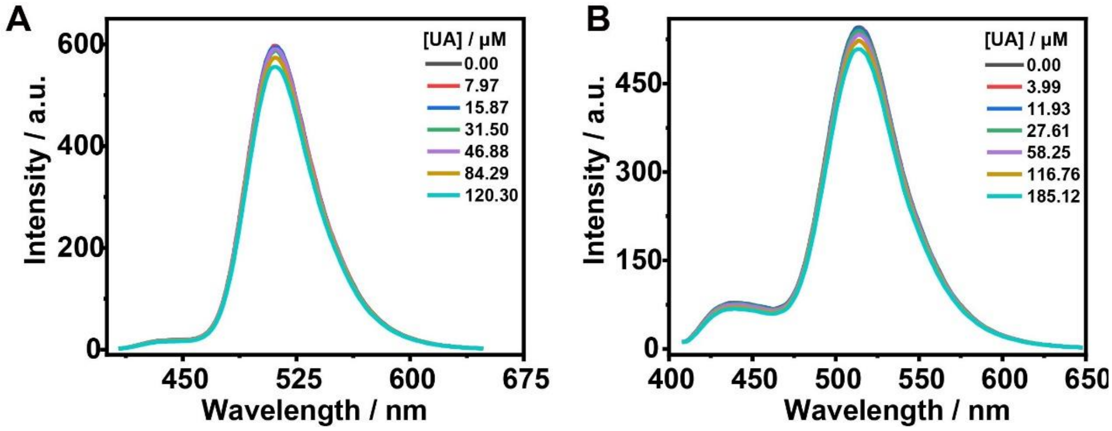

Figure S7. Fluorescence spectra of HPTS with UA and competitive titration of the *γ*-CD•HPTS reporter pair with UA in 10 mM HEPES buffer solution (pH = 7.4, 25 °C) at λ_{ex} = 405 nm and λ_{em} = 435 nm. (A) Fluorescence spectra of HPTS (10.00 μM) by adding UA up to 120.30 μM. (B) Competitive titration of the *γ*-CD•HPTS (10.00 mM/10.00 μM) reporter pair with UA up to 185.12 μM.

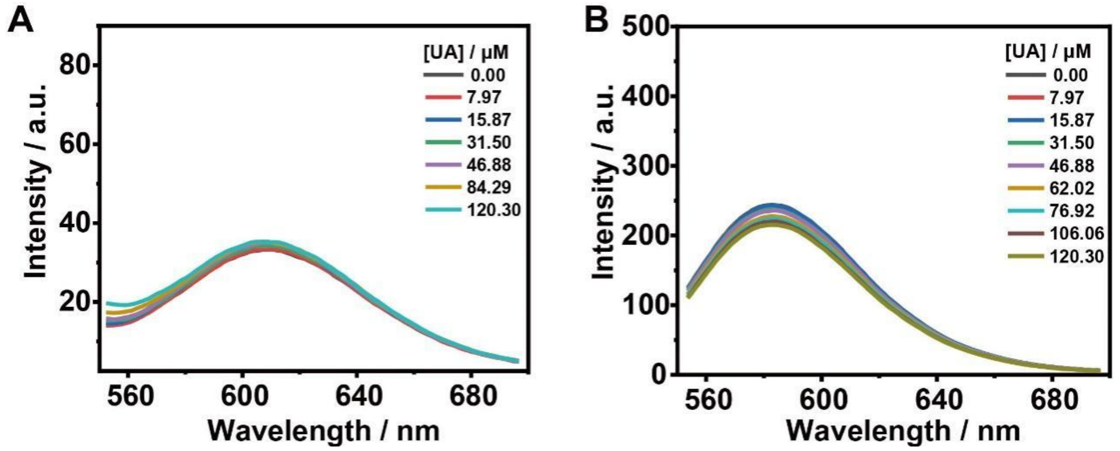

Figure S8. Fluorescence spectra of DSMI with UA and competitive titration of the CB[6]•DSMI reporter pair with UA in 10 mM HEPES buffer solution (pH = 7.4, 25 °C) at λ_{ex} = 450 nm and λ_{em} = 582 nm. (A) Fluorescence spectra of DSMI (1.00 µM) by adding UA up to 120.30 µM. (B) Competitive titration of the CB[6]•DSMI (8.00/1.00 µM) reporter pair with UA up to 120.30 µM.

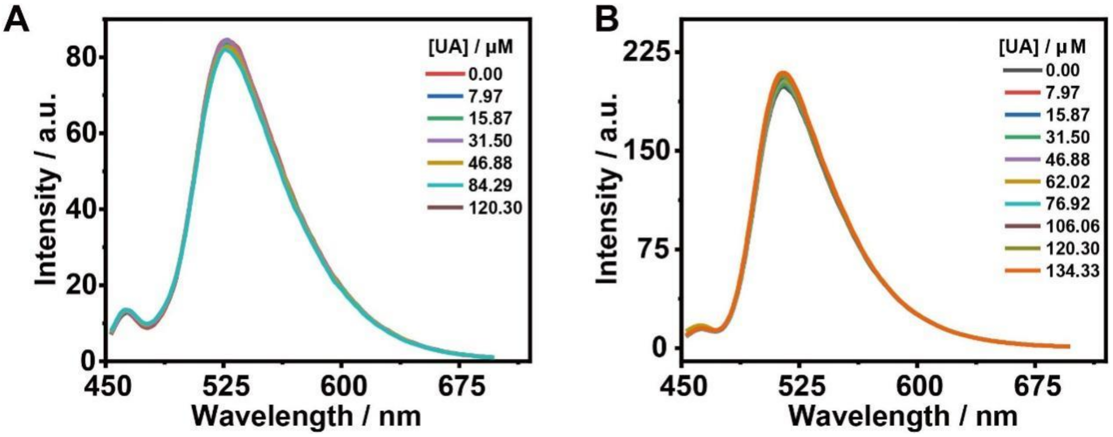

Figure S9. Fluorescence spectra of AO with UA and competitive titration of the CB[7]•AO reporter pair with UA in 10 mM HEPES buffer solution (pH = 7.4, 25 °C) at λ_{ex} = 450 nm and λ_{em} = 510 nm. (A) Fluorescence spectra of AO (0.50 μM) by adding UA up to 120.30 μM. (B) Competitive titration of the CB[7]•AO (15.00/0.50 μM) reporter pair with UA up to 134.33 μM.

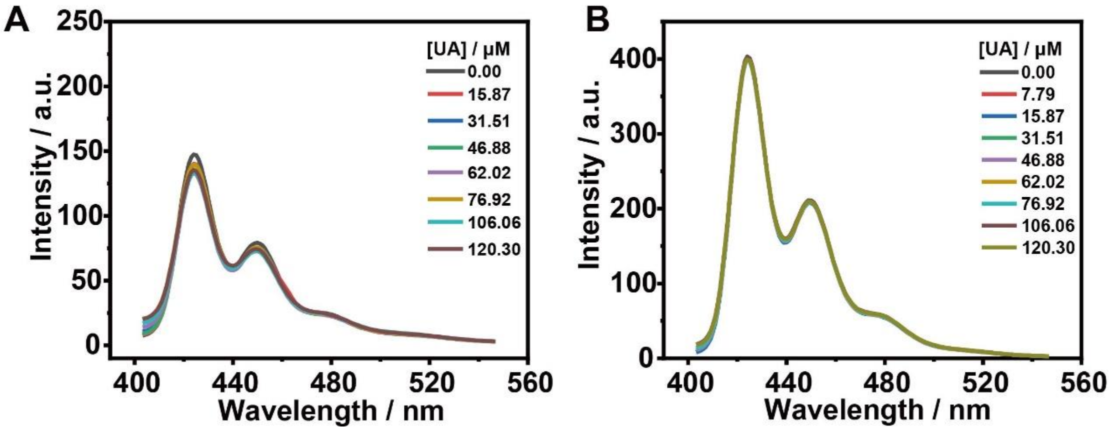

Figure S10. Fluorescence spectra of Me_2DAP with UA and competitive titration of the CB[8]•Me_2DAP reporter pair with UA in 10 mM HEPES buffer solution (pH = 7.4, 25 °C) at λ_{ex} = 335 nm and λ_{em} = 449 nm. (A) Fluorescence spectra of Me_2DAP (1.00 μM) by adding UA up to 120.30 μM. (B) Competitive titration of the CB[8]•Me_2DAP (2.00/1.00 μM) reporter pair with UA up to 120.30 μM.

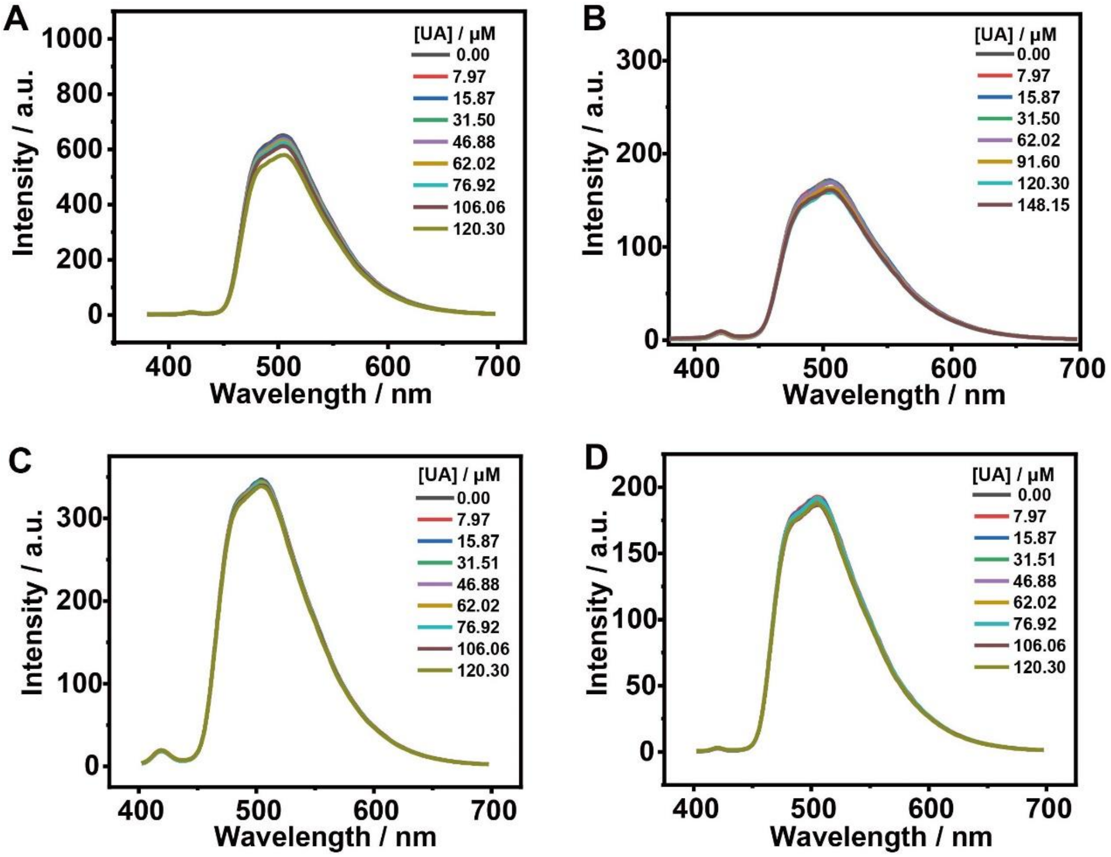

Figure S11. Fluorescence spectra of LCG with UA and competitive titration of the SC*n*As•LCG reporter pairs with UA in 10 mM HEPES buffer solution (pH = 7.4, 25 °C) at λ_{ex} = 368 nm and λ_{em} = 505 nm. (A) Fluorescence spectra of LCG (0.50 μM) by adding UA up to 120.30 μM. (B) Competitive titration of the SC4A•LCG (0.50/0.50 μM) reporter pair with UA up to 148.15 μM. (C) Competitive titration of the SC5A•LCG (1.00/1.00 μM) reporter pair with UA up to 120.30 μM. (D) Competitive titration of the SC6A•LCG (0.25/1.00 μM) reporter pair with UA up to 120.30 μM.

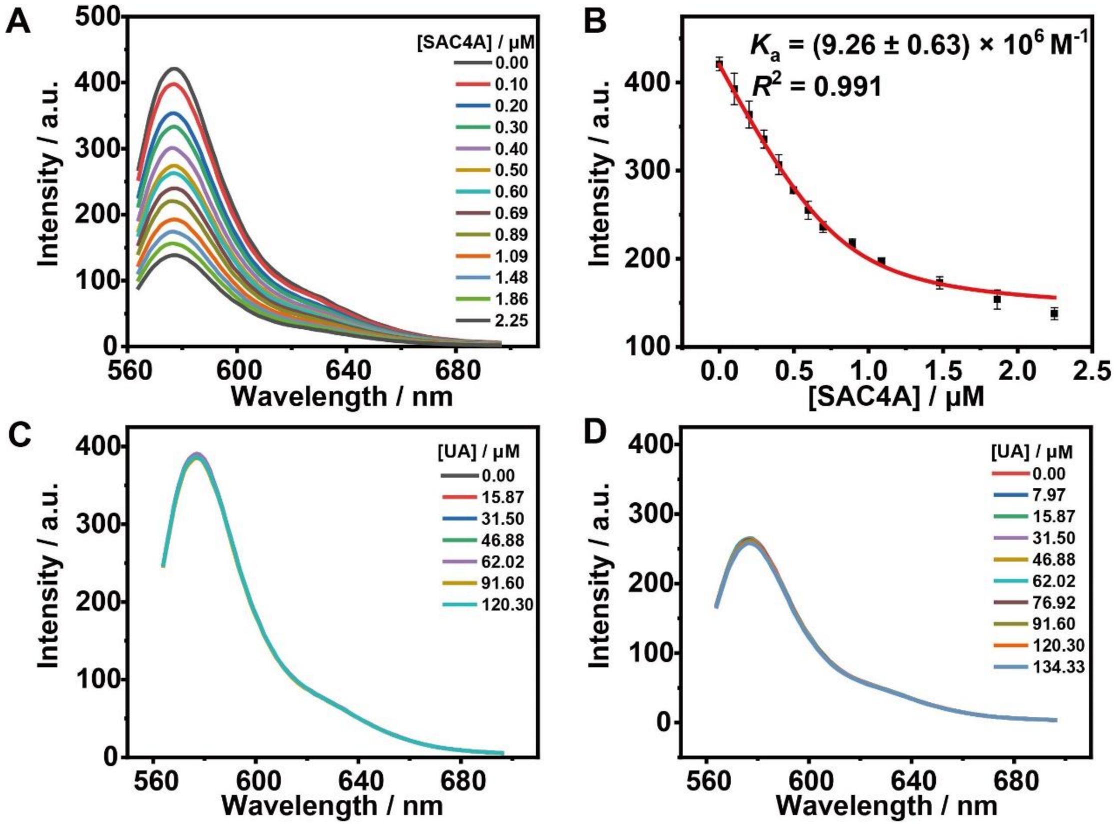

Figure S12. Direct fluorescence titration of RhB with SAC4A, fluorescence spectra of RhB with UA, and competitive titration of the SAC4A•RhB reporter pair with UA in 10 mM HEPES buffer solution (pH = 7.4, 25 °C) at λ_{ex} = 554 nm and λ_{em} = 576 nm. (A) Direct fluorescence titration of RhB (0.80 μM) with SAC4A up to 2.25 μM. (B) The titration curve at λ_{em} = 576 nm resulted from a 1:1 binding model. (C) Fluorescence spectra of RhB (0.80 μM) by adding UA up to 120.30 μM. (D) Competitive titration of the SAC4A•RhB (1.00/0.80 μM) reporter pair with UA up to 134.33 μM.

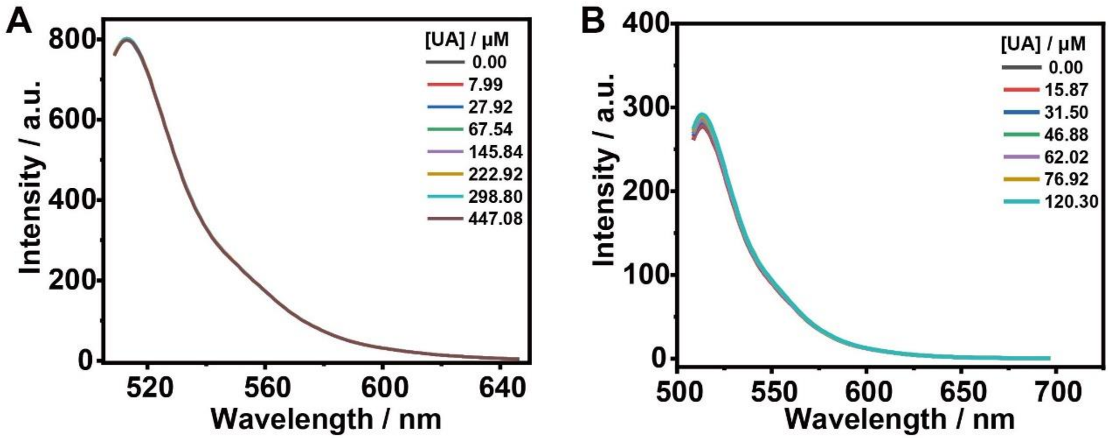

Figure S13. Fluorescence spectra of Fl with UA and competitive titration of the GC4A-4C•Fl reporter pair with UA in 10 mM HEPES buffer solution (pH = 7.4, 25 °C) at λ_{ex} = 500 nm and λ_{em} = 513 nm. (A) Fluorescence spectra of Fl (1.00 μM) by adding UA up to 447.08 μM. (B) Competitive titration of the GC4A-4C•Fl (8.00/1.00 μM) reporter pair with UA up to 120.30 μM.

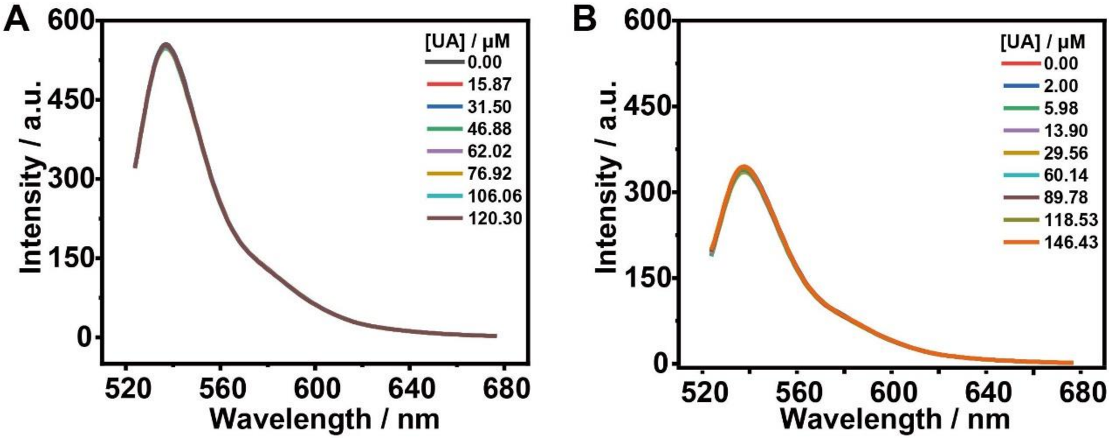

Figure S14. Fluorescence spectra of EY with UA and competitive titration of the GC4AOEG•EY reporter pair with UA in 10 mM HEPES buffer solution (pH = 7.4, 25 °C) at λ_{ex} = 517 nm and λ_{em} = 537 nm. (A) Fluorescence spectra of EY (0.50 μM) by adding UA up to 120.30 μM. (B) Competitive titration of the GC4AOEG•EY (4.00/0.50 μM) reporter pair with UA up to 146.43 μM.

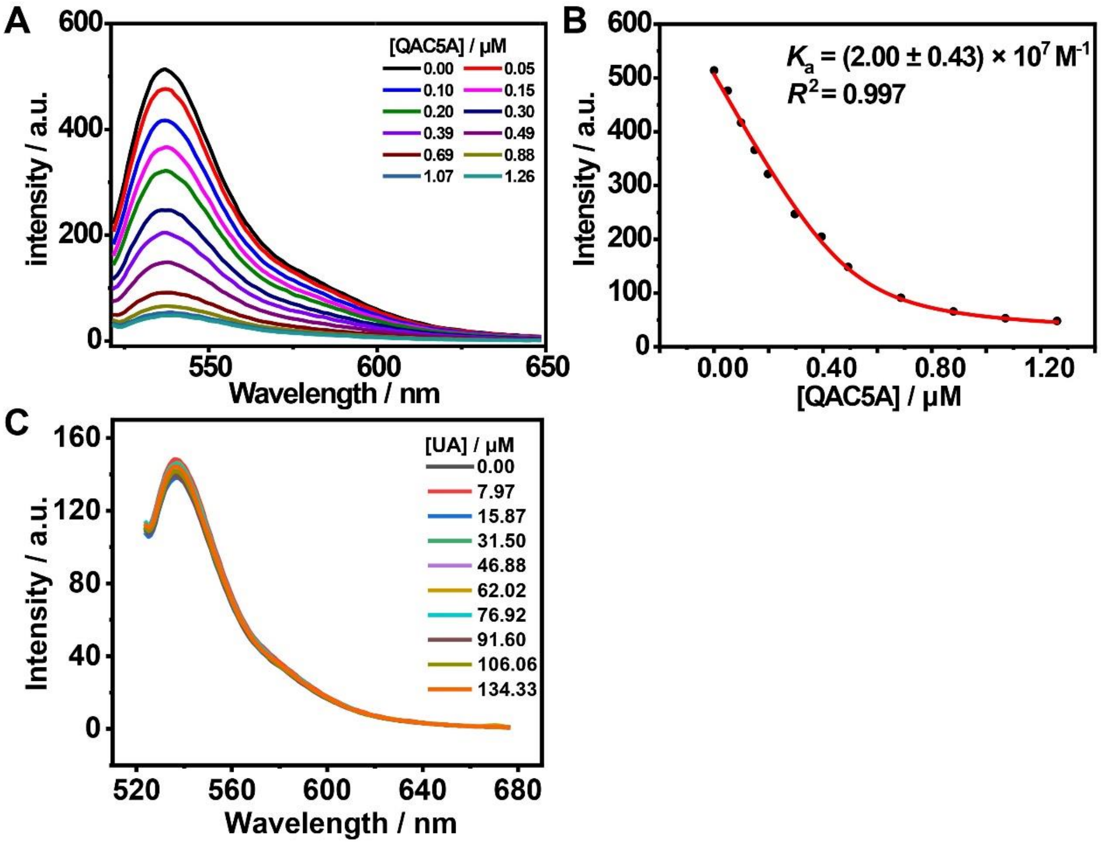

Figure S15. Direct fluorescence titration of EY with QAC5A and competitive titration of the QAC5A•EY reporter pair with UA in 10 mM HEPES buffer solution (pH = 7.4, 25 °C) at λ_{ex} = 517 nm and λ_{em} = 537 nm. (A) Direct fluorescence titration of EY (0.5 μM) with QAC5A up to 1.26 μM. (B) The titration curve at λ_{em} = 537 nm acquired by a 1:1 binding model. (C) Competitive titration of the QAC5A•EY (0.40/0.50 μM) reporter pair with UA up to 134.33 μM.

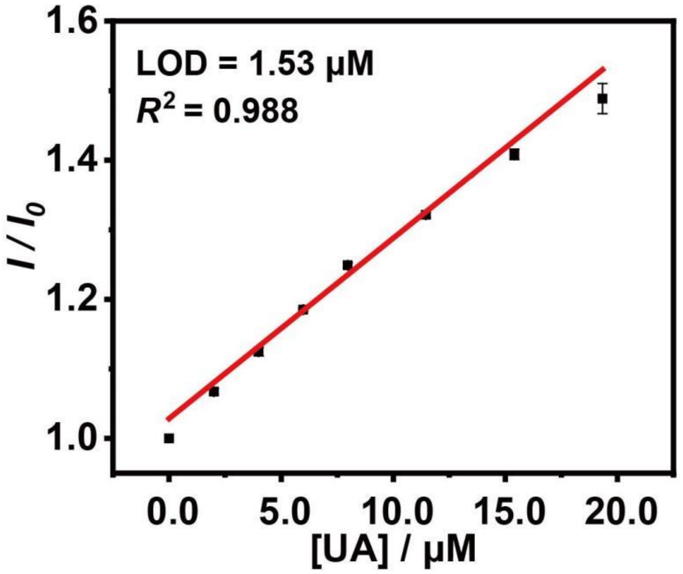

Figure S16. The limit of detection (LOD) for UA in HEPES buffer solution (10 mM, pH 7.4). The plot of I/I_0 against UA concentration in 10 mM HEPES buffer solution (pH = 7.4, 25 °C) with I_0 and I assigned as the fluorescence intensities of the GC5A•Fl (0.80/1.00 μM) reporter pair in the absence and presence of UA (0 – 19.33 μM), respectively.

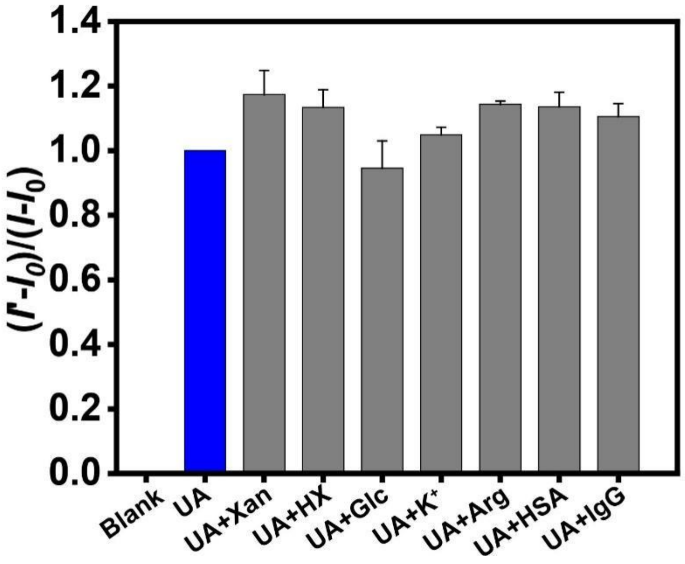

Figure S17. Fluorescence responses of GC5A•Fl (0.80/1.00 μM) reporter pair at λ_{ex} = 500 nm and λ_{em} = 513 nm upon addition of UA (10 μM) and endogenous substances, including (0.40 mg/L for HSA, 5 mg/L for IgG, 3 mM for Glc, 0.30 mM for K^+ and Arg, and 10 μM for Xan and HX, respectively) in HEPES buffer solution (10 mM, pH = 7.4) at 25 °C (n = 3). I_0 and I are the fluorescence intensities of the GC5A•Fl complex (0.80/1.00 μM) in the absence and presence of UA, I' represents the fluorescence intensity of the GC5A•Fl complex (0.80/1.00 μM) caused by UA and endogenous substances, respectively.

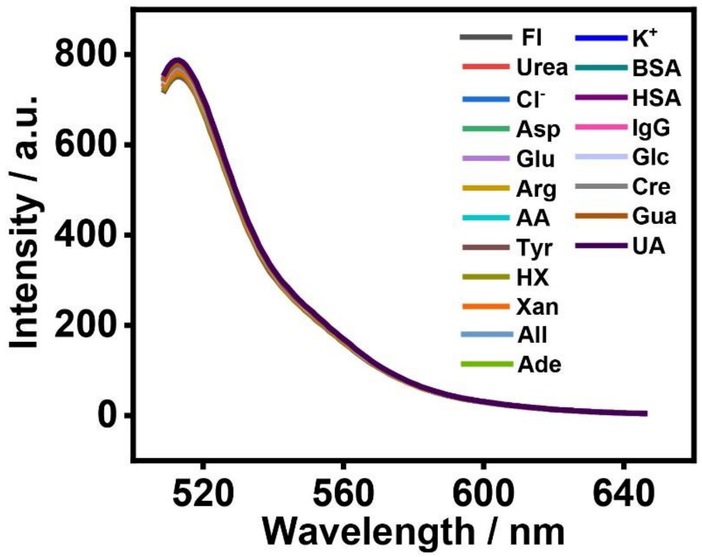

Figure S18. Fluorescence spectra of Fl (1.0 μM) by the cumulative addition of endogenous substances (0.40 mg/L for BSA and HSA, 5 mg/L for IgG, 3 mM for Glc, 0.30 mM for urea, Cl^-, K^+, Tyr, Arg, Asp and Glu, and 10 μM for UA, Xan, HX, All, Ade, Gua and Cre, respectively) in HEPES buffer solution (10 mM, pH = 7.4) at 25 °C.

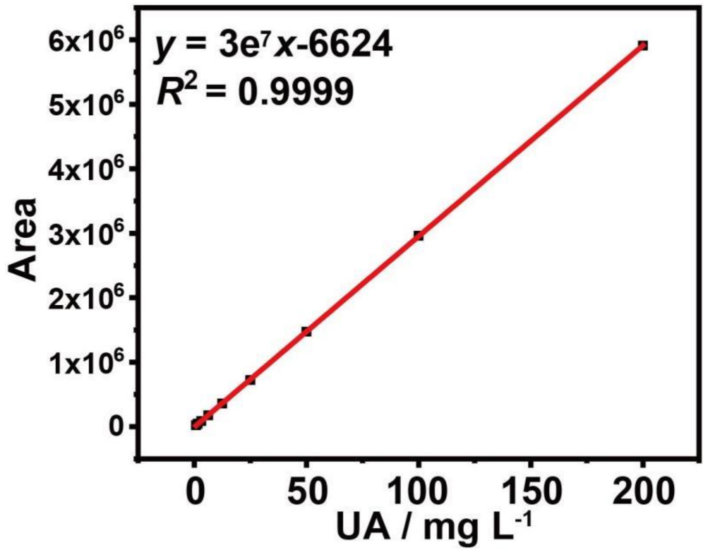

Figure S19. The correlation between peak area and the increased level of UA (0.78 – 200.00 mg L^{-1}) by HPLC method (R^2 = 0.9999).
